# Genomic Diversity within the *Enterobacter cloacae* Complex

**DOI:** 10.1371/journal.pone.0003018

**Published:** 2008-08-21

**Authors:** Armand Paauw, Martien P. M. Caspers, Frank H. J. Schuren, Maurine A. Leverstein-van Hall, Alexis Delétoile, Roy C. Montijn, Jan Verhoef, Ad C. Fluit

**Affiliations:** 1 Department of Medical Microbiology, University Medical Centre Utrecht, Utrecht, The Netherlands; 2 TNO Department of Microbiology, Zeist, The Netherlands; 3 Unité Biodiversité des Bactéries Pathogènes Emergentes, Institut Pasteur, Paris, France; University of British Columbia, Canada

## Abstract

**Background:**

Isolates of the *Enterobacter cloacae* complex have been increasingly isolated as nosocomial pathogens, but phenotypic identification of the *E. cloacae* complex is unreliable and irreproducible. Identification of species based on currently available genotyping tools is already superior to phenotypic identification, but the taxonomy of isolates belonging to this complex is cumbersome.

**Methodology/Principal Findings:**

This study shows that multilocus sequence analysis and comparative genomic hybridization based on a mixed genome array is a powerful method for studying species assignment within the *E. cloacae* complex. The *E. cloacae* complex is shown to be evolutionarily divided into two clades that are genetically distinct from each other. The younger first clade is genetically more homogenous, contains the *Enterobacter hormaechei* species and is the most frequently cultured *Enterobacter* species in hospitals. The second and older clade consists of several (sub)species that are genetically more heterogonous. Genetic markers were identified that could discriminate between the two clades and cluster 1.

**Conclusions/Significance:**

Based on genomic differences it is concluded that some previously defined (clonal and heterogenic) (sub)species of the *E. cloacae* complex have to be redefined because of disagreements with known or proposed nomenclature. However, further improved identification of the redefined species will be possible based on novel markers presented here.

## Introduction

*Enterobacter cloacae* is a facultative anaerobic Gram-negative bacillus belonging to the family of *Enterobacteriaceae*. The nomenclature (taxonomy) of the *E. cloacae* complex is mainly based on whole genome DNA-DNA hybridizations and phenotypic characteristics [1]–[3]. Currently, 6 species have been assigned to the *Enterobacter cloacae* complex, including *Enterobacter asburiae*, *Enterobacter cloacae*, *Enterobacter hormaechei*, *Enterobacter kobei*, *Enterobacter ludwigii*, and *Enterobacter nimipressuralis*. Only *Enterobacter* isolates that belong to the *E. cloacae* complex are of clinical significance and are increasingly isolated as nosocomial pathogens [4], [5]. In surveillance studies, *Enterobacter* species are often not further classified beyond the genus level probably because identification is difficult. *Enterobacter* spp. causes 7% of nosocomial infections in intensive care units in the USA [6], [7].

Accurate species identification is desirable for determining whether specific species within the *E. cloacae* complex are more prone to cause infections. More precise identification of *E. cloacae* complex isolates may permit differentiation between nosocomial species and commensal or transitional species. However, until now phenotypic identification of species and subspecies within the *E. cloacae* complex have been largely unreliable and irreproducible [8]. In this study, the discriminatory power of four genetically-based approaches was evaluated. These methods were *hsp60*- and *rpoB*-genotyping, multi-locus sequence analysis (MLSA), and comparative genomic hybridization (CGH).

The first method entailed genotypic identification via sequencing of a fragment of the heat shock protein 60 gene (*hsp60*), and was included because *hsp60* genotyping appeared to be a promising novel method. Using this approach, the *E. cloacae* complex was divided into 12 genetic clusters (I–XII) and an unstable sequence cluster (XIII),(Table 1) [9].

**Table 1:** The different clusters of the *Enterobacter cloacae* complex and their nomenclature, according *hsp60* genotyping by Hoffmann and Roggenkamp [9].

Clusters	Species	Reference
I	*E. asburiae*	[2]
II	*E. kobei*	[41]
III	*E. cloacae* III	[9]
IV	*E. cloacae* IV	[9]
V	*E. ludwigii*	[42]
VI	*E. hormaechei* subsp. *oharae*	[43]
VII	*E. hormaechei* subsp. *hormaechei*	[43]
VIII	*E. hormaechei* subsp. *steigerwaltii*	[43]
IX	*E. cloacae* IX	[9]
X	*E. nimipressuralis*	[2]
XI	*E. cloacae* subsp. *cloacae*	[4]
XII	*E. cloacae* subsp. *dissolvens*	[4]
xiii	*E. cloacae* sequence crowd	[9]

The second method consisted of sequencing a fragment of *rpoB*, and possibly represents a good alternative for strain identification because of the high resolution available to differentiate between closely related species [10].

The third approach employed MLSA, a more sophisticated technique. With this method, several widely separated genomic loci are analyzed, which minimizes the effect of recombination events on clustering analysis.

Finally, microarray-based CGH was also used; this approach is a powerful method for performing genome-wide studies on different bacteria [11]–[19].

The *E. cloacae* complex could thus be divided into two genetically distinct clades that were not previously recognized. The younger first clade contains the *E. hormaechei* species. This species is possibly more clinically relevant because it represents the most frequently cultured *Enterobacter* species in hospitals. The second clade of the *E. cloacae* complex consists of several clonal and heterogeneous (sub)species, leading us to propose a redefinition of species assignment within this genomically diverse complex.

## Results

### Identification of isolates based on *hsp60* sequences

The population structure of the *E. cloacae* complex in hospitals was studied using 158 isolates. Of these, 120 (including 27 outbreak I isolates and 13 commensal fecal isolates) were obtained in the University Medical Centre Utrecht, Utrecht, The Netherlands (UMCU); 21 isolates (including 10 outbreak I isolates) from 11 other Dutch hospitals; 12 isolates from 12 different European hospitals; 3 isolates from an industrial site, and 2 isolates were *E. cloacae* ATCC13047. (See Materials and Methods for more information about the isolates). All isolates were identified as *E. cloacae* using the Phoenix Automated Microbiology System (Becton Dickinson Biosciences, Sparks, MD, U.S.). These results were compared to identification based on sequencing of a previously described 273 bp fragment of *hsp60* that appeared to give reliable identification of *E. cloacae* complex isolates [9]. Twenty-nine different sequences were obtained and compared with sequences described by Hoffman and Roggenkamp [9]. The isolates were identified as *E. cloacae* III (n = 26), *E. cloacae* IV (n = 13), *E. cloacae* IX (n = 1), *E. cloacae cloacae* (n = 4), *E. cloacae dissolvens* (n = 1), *E. hormaechei oharae* (n = 13), *E. hormaechei steigerwaltii* (n = 78), *Enterobacter kobei* (n = 7), *E. ludwigii* (n = 6), and *E. asburiae* (n = 9) (Table S1). The *hsp60* genotyping data indicate that phenotypic identification using the Phoenix Automated Microbiology System cannot discriminate between different species and subspecies of the *E. cloacae* complex.

### Identification of isolates based on *rpoB* sequences

Because identification of *Enterobacteriaceae* by sequencing of a fragment of *rpoB* has been reported as a robust and promising method for identification [19], isolates were also classified based on the *rpoB* sequence. Thirty-two different sequences were detected in the set of 158 isolates after analysis of a 501 bp fragment of *rpoB*. Sequences were compared with a database of *rpoB* sequences that represents the major phylogenetic clades in the family *Enterobacteriaceae* [20]; this database is managed by the Pasteur Institute in Paris, France. The isolates were identified as *E. hormaechei* (n = 117), *Enterobacter asburiae* (n = 10), *E. cloacae* (n = 5), *Enterobacter cloacae* IV (n = 20), and *Enterobacter ludwigii* (n = 6) (Table S1). As with *hsp60* genotyping, the results of *rpoB* typing showed that phenotypic identification by the Phoenix Automated Microbiology System cannot discriminate between different species and subspecies of the *E. cloacae* complex. Furthermore, the results also show discrepancies between the two single gene genotyping methods (Table S1). These discrepancies may at least in part be explained by recombination events.

### Reliability of DNA fragments for MLSA analysis of the *Enterobacter cloacae* complex

To minimize the effects of potential recombination events between isolates, MLSA was used. Gene fragments of five additional genes (*fusA*, *gyrB*, *leuS*, *pyrG*, and *rplB*) of 50 *E. cloacae* complex isolates were sequenced. These isolates where selected based on their differences in *hsp60* sequence and a preliminary analysis of the CGH data. For unique *hsp60* sequence, one isolate was selected. In addition, isolates with similar *hsp60* sequences but different preliminary CGH clustering results were included with the limitation that only one isolate was selected from each cluster (data not shown). The accession numbers of the sequenced gene fragments are listed in Table S2. Initial analysis of the sequence data revealed that the *E. cloacae* complex consists of 2 clades. To determine whether the sequence data was appropriate for phylogenetic studies, we assessed whether positive selection might have affected our data using two population genetic tests: Tajima's D statistic [21] and Fu's F_s_ statistic [22], [30] (Table 2). Tajima's D statistic only produced a significant (p<0.05) result for the *rplB* gene from clade 1. This indicates that the *rplB* gene might be under positive selection. This finding is supported by a significant result in Fu's F test (p<0.01). In addition, the transition/transversion ratio of *rplB* was estimated by plotting the transversion and transition rates as a function of the genetic distances (data not shown). The data show no saturation for either curve. However, the transition/transversion ratio was estimated to be 1, indicating a non-random distribution of mutation frequency. This supports the notion that the *rplB* gene is under positive selection. The only other significant Fu's F test was for the *rpoB* fragment from clade 2, indicating that these isolates are possibly under positive selection or that this population expands. However, this result was not supported by Tajima's D test (Table 2) or the K_a_/K_s_ ratio (data not shown). Therefore, positive selection of *rpoB* is not likely. These results indicate that all gene fragments except for *rplB* are suitable for phylogenetic analysis of the *E. cloacae* complex.

**Table 2:** Polymorphisms observed in seven protein-encoding genes among 50 *E. cloacae* complex isolates.

Clade 1 (n = 30)	Length (bp)	No of variable sites	no. of haplotypes	π				Tajima's D	Fu's Fs
				θ (Total)	Total	synonymous changes	non-synonymous changes		
included for analysis									
*fusA*	633	11	9	0.0044	0.0039	0.0153	0.0006	−0.3246	−1.339
*gyrB*	417	43	18	0.0297	0.0284	0.0910	0.0075	−0.1594	−1.437
*leuS*	642	54	19	0.0232	0.0201	0.0817	0.0008	−0.5045	−1.761
*pyrG*	306	8	8	0.0066	0.0056	0.0227	0.0000	−0.4510	−1.817
*rpoB*	501	21	14	0.0106	0.0089	0.0351	0.0000	−0.5739	−3.083
*hsp60*	273	26	10	0.0259	0.0234	0.0842	0.0022	−0.0351	1.387
Mean	2772	163	25	0.0160	0.0143^a^	0.0538	0.0017	−0.4061	−1.391
excluded from further analyses									
*rplB*	333	4	6	0.0038	0.0012	0.0043	0.0000	−1.87843^b^	−4.834^c^
Clade 2 (n = 20)									
included for analysis									
*fusA*	633	21	11	0.0098	0.0087	0.0319	0.0018	−0.4267	−1.364
*gyrB*	417	90	19	0.0737	0.0671	0.2241	0.0141	−0.3705	−3.526
*leuS*	642	157	20	0.0053	0.0947	0.3399	0.0175	0.1523	−2.776
*pyrG*	306	32	15	0.0350	0.0418	0.1476	0.0074	0.7711	−1.783
*rpoB*	501	32	18	0.0191	0.0173	0.0686	0.0000	−0.0384	−8.191^c^
*hsp60*	273	44	17	0.0578	0.0507	0.1719	0.0079	−0.5243	−3.707
Mean	2772	376	20	0.0475	0.0467^a^	0.1663	0.0082	−0.0700	−1.116
excluded from further analyses									
*rplB*	333	9	7	0.0076	0.0097	0.0357	0.0000	0.9347	0.229

a Difference indicates that clade 2 is older than clade 1.

b P<0.05.

c P<0.01.

**Figure 1:**
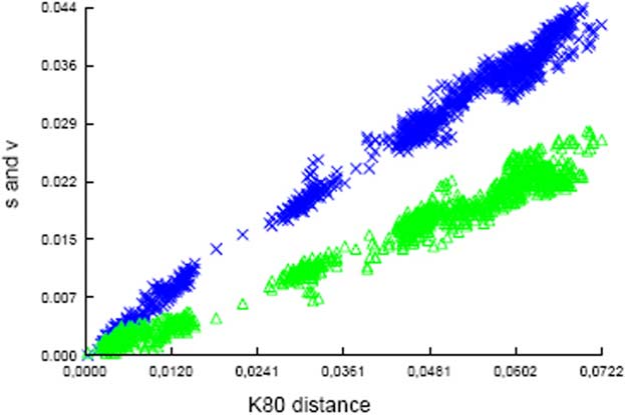
Transitions/transversion vs genetic distance. From the six concatenated sequences used for phylogentic studies the transitions (blue crosses) and transversions (green triangles) are plotted against genetic distance according to the K80 model. No saturation is detected and the transitions/transversion ratio is estimated at 2.

The quality of the phylogenetic information obtained for the other remaining genes was also analyzed by plotting the transversion and transition rates as a function of the genetic distance (Figure 1). Transversion and transition rates increase concomitantly with genetic distance, indicating that there is little or no saturation. The transition/transversion ratio was estimated to be 2. These data further support that the sequences of the remaining genes can be used for phylogenetic studies.

### Phylogenetic analyses

**Figure 2:**
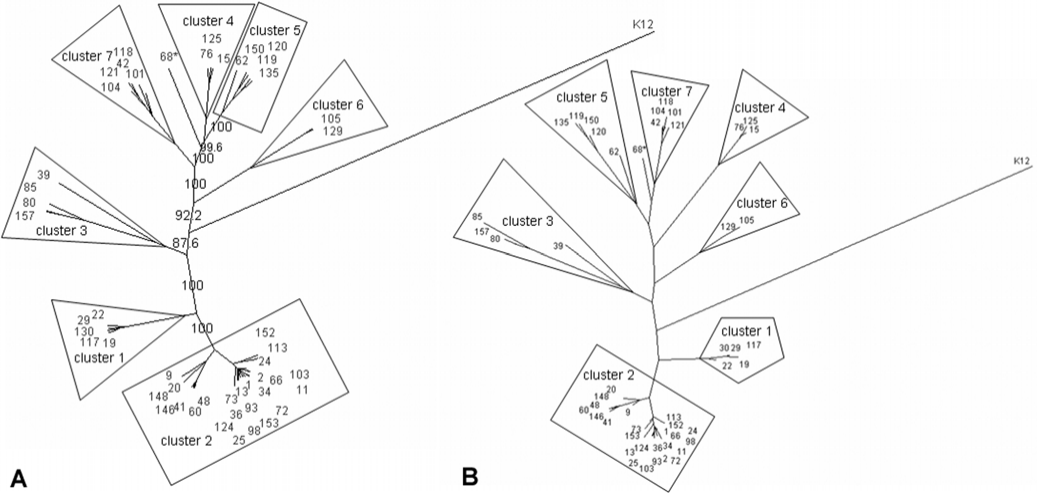
Phylogenetic trees of *E. cloacae* complex. A) Phylogenetic tree based on results of MLSA clustered with Clonalframe. Numbers indicate confidence values of the branches. * Solitaire isolate. K12: *E. coli* K12 used as the outgroup. B) Phylogenetic tree based on concatenated sequences of six gene fragments clustered with the Maximum Likelihood. Each color depicts a cluster according to the CGH results. * Solitaire isolate. K12: *E. coli* K12 used as the outgroup.

The genetic heterogeneity of the *E. cloacae* complex was analyzed by generating phylogenetic trees based on the MLSA data. Noncongruence between trees based on the sequences of single DNA fragments indicated that recombination events had occurred (data not shown) [23]. Genetic relationships between the isolates could not be determined by E-burst or minimal spanning tree, because the concatenated sequences of the six gene fragments contained more than two mutations per sequence. Therefore, ClonalFrame was used to determine the genetic relationship between the tested isolates. The resulting tree contained the sequences of 51 isolates, including 50 *E. cloacae* complex isolates and one *Escherichia coli* K12 that was used as the outgroup (Figure 2A). For reasons mentioned previously, the sequences of the *rplB* gene were not included in the final analysis. The nodes of several branches were different when using maximum likelihood clustering with concatenated sequences of the six gene fragments (Figure 2B). However, isolates that clustered together in the same branches of the ClonalFrame tree also clustered in the maximum likelihood-tree. This indicates that the genetic relationship between the clusters is perhaps uncertain but that isolates in each cluster are genetically similar to each other because the same isolates cluster together in both trees. The recombination/mutation ratio was 1.04 (95% confidence interval 0.72–1.45), indicating that recombination events are involved in the evolution of the *E. cloacae* complex. This may explain the differences between the phylogenetic trees. Based on the ClonalFrame tree the isolates, except one were divided into 7 separate clusters (Figure 2A+B).

### Comparative genome hybridization analysis

The six DNA fragments used for MLSA present only a small fraction of the genome. To evaluate whether MLSA results are representative of population structure on the genomic level, comparative genome hybridization (CGH) was performed. CGH was performed with 3,072 DNA fragments from a shotgun library obtained from 8 *E. cloacae* complex isolates (7 strains); these fragments functioned as probes (mixed genome array or MGA). After analyzing all 158 slides, a total of 2,614 spots (85%) met the quality criteria and were included in the study (Table S3). The average size of the DNA fragments spotted on the slides was 1202 bp. Eighteen randomly selected DNA fragments of the core genome (core genome was defined as genes present in all isolates with a likelihood of >95%) were sequenced (Table S4). The average size of fragments representing the core genome was 1178 bp. No redundancy was detected in these sequenced DNA fragments. The overall redundancy of the MGA was calculated to be less than 5.3%, thus a maximum of 138 of the 2,614 DNA fragments have a sequence overlap with another insert. The total genetic coverage of each of the seven different strains used for construction of the array was calculated [16], [24] (Table 3). The gene coverage varied between 55% and 60% for the 7 isolates and the pan-genomic coverage of the *E. cloacae* complex was estimated to be 55% to 60%. The calculated minimal nucleotide coverage of the core genome was 58%. Results show that more than half of the genes, but also non-encoding or other coding DNA sequences, are represented in the constructed MGA. For inserts EnCl.014E12 and EnCl.020H03, 98% and 97% of the PCR results correlated with the results of the CGH array, respectively. The high similarity between CGH and PCR results indicate that MGA hybridization results were consistent. All replicates were found in the same cluster as the originals. Clearly, CGH array data are reliable for comparative genomic studies.

**Table 3:** Characteristics of isolates used for construction of DNA library and the calculated gene coverage of each isolate.

Isolate no.	Country	Hospital	Year	Source	cluster^a^	*rpoB* genotyping	*hsp60* genotyping	PFGE-type	gene coverage (%+/−SD^b^)	plasmid pQC
31	NL	1	2002	wound/sst^c^	2	*E. hormaechei*	*E. hormaechei steigerwaltii*	1	58.5+/−2.2	positive
55	NL	1	2003	rectal swab	2	*E. hormaechei*	*E. hormaechei steigerwaltii*	1	58.5+/−2.2	positive
48	NL	1	2002	urine tract	2	*E. hormaechei*	*E. hormaechei oharae*	14	56	negative
19	NL	1	2003	pneumonia	1	*E. hormaechei*	*E. cloacae* III	15	60+/−4	negative
142	AUS	16	1997	wound/sst	1	*E. hormaechei*	*E. cloacae* III	17	58	negative
146	ITA	20	1998	blood	2	*E. hormaechei*	*E. hormaechei oharae*	27	60	negative
104	NL	1	2003	rectal swab	7	*E. cloacae* IV	*E. cloacae* IV	53	56+/−3	positive
153	POL	25	1998	pneumonia	2	*E. hormaechei*	*E. hormaechei steigerwaltii*	56	62	negative

a Cluster according MLSA results.

b If replicates are tested the standard deviation is calculated.

c Wound or skin or soft tissue infection. Abbreviations, Aus; Austria, ITA; Italy, NL; The Netherlands, and POL; Poland.

**Figure 3:**
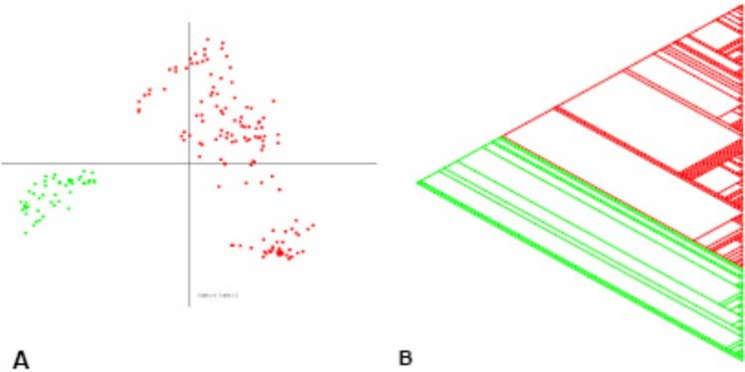
Analyze of the comparative genome hybridization results. Comparative genome hybridization data clustered with PCA and maximum parsimony. Comparison of CGH data of 180 slides tested for 1150 DNA fragments A) with PCA; B) with the Parsimony method. Both figures show that isolates of the *E. cloacae* complex are divided in two separate clades. Red: first clade with 116 different isolates; Green: second clade with 42 different isolates.

**Figure 4:**
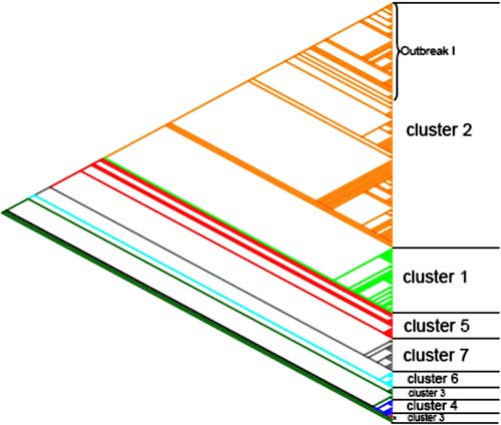
Tree constructed on basis of the CGH results of the Parsimony method. The slanted cladogram depicts genetic relationships between isolates each color represents a different MLSA cluster. *Solitaire isolate in MLSA.

Of the 2,614 spots analyzed, 1,358 were considered to contain core genome DNA fragments because they were present in all isolates tested. These fragments were omitted for further analysis because they do not contribute to the speciation of the *E. cloacae* complex. Principal component analysis (PCA) showed that the isolates were mainly divided on the basis of the absence or presence of the pQC plasmid (data not shown). Based on the PCA data and because pQC is approximately 300 kb in size, it was expected that pQC-derived DNA fragments in the MGA would influence the assignment of isolates to different species or clades. Statistical and clustering analysis (described in Materials and Methods) showed that 106 DNA fragments were likely derived from the pQC plasmid. These 106 plasmid-derived DNA fragments were removed from further analysis. PCA of the hybridization results of the remaining 1,150 DNA fragments showed that the *E. cloacae* complex is divided into two genetically distinct clades (Figure 3A). Subsequently, these results where confirmed with a phylogenetic tree created with hierarchical clustering (not shown). Using the Pars program of the PHYLIP software package, a parsimony tree was computed (Figure 3B) that confirmed the genetic distinction between the two clades. Isolates that were not previously typed by MLSA were clustered based on the results of the constructed parsimony tree (Figure 4). Clusters 1–2 represent isolates from the first clade, whereas isolates from the second clade belong to clusters 3–7 and the solitaire isolate. Clusters 1–7 contained 25, 91, 6, 6, 9, 7, and 13 isolates, respectively.

### Comparison of the results of the different typing techniques

Cluster 1 isolates were identified as *E. hormaechei* using *rpoB* sequencing, but according to *hsp60* genotyping, all isolates were *E. cloacae* III (Table S1). This cluster was a separate branch in the first clade of both trees on CGH and MLSA results. Cluster 2 isolates were all *E. hormaechei* isolates according to *rpoB*, but *E. hormaechei steigerwaltii* and *E. hormaechei oharae* according to *hsp60* genotyping. These isolates were highly similar according to MLSA, but genetically heterogeneous according to CGH. MLSA-based further subdivision of cluster 2 was not reliable. However, the tree based on CGH results showed that the outbreak I isolates consisted of a separate branch in cluster 2. This clonality of outbreak I isolates is also supported by the similarity of their *rpoB* and *hsp60* sequences. The results from the second clade were less congruent in the CGH tree. Cluster 5 isolates formed a distinct cluster in CGH and MLSA and were all identified as *E. asburiae* by *rpoB* and *hsp60*. Cluster 4 isolates were all genotyped as *E. ludwigii* and clustered together both in MLSA and CGH. Cluster 6 as well as cluster 7 were separate clusters in MLSA and CGH but all belonged to *E. cloacae* IV according to *rpoB* genotyping, whereas according to *hsp60* genotyping all cluster 7 isolates were *E. cloacae* IV and all cluster 6 isolates were *E. kobei*. Isolates from cluster 3 were mixed on CGH analysis, indicating a heterogeneous group consisting of multiple species. The solitaire isolate belonged to *E. asburiae* according to *rpoB* but *E. cloacae* III according to *hsp60*. Furthermore, this isolate also clustered separately in the CGH tree and the maximum likelihood tree, indicating that this specific isolate may represent yet another species.

Except for a lack of congruence with cluster 3 the results of CGH and MLSA were identical. This indicates that MLSA results are also highly representative of genomic relationships between *E. cloacae* complex isolates. However, the findings with CGH or MLSA are inconsistent with those obtained using fragments of *rpoB* or *hsp60* (Table S1).

### Evolution of the *Enterobacter cloacae* complex

The mean pairwise nucleotide diversity (π) of the 50 *E. cloacae* complex isolates was higher within clade 2 than clade 1 (0.047+/−0.003 vs. 0.014+/−0.002), suggesting that isolates from clade 2 are older than isolates from clade 1. Furthermore, the number of alleles was relatively lower in clade 1 compared to clade 2 (25/30 vs 20/20), which supports this finding (Table 2).

### Selective markers

MLSA and CGH are costly and time-consuming techniques, whereas effective patient therapy requires inexpensive and rapid results. Therefore, new approaches that rapidly identify isolates are in demand. Genetic markers specific for each species may facilitate quick and inexpensive tests. Specific markers were identified within the set of 1,150 genes that were variably present among the *E. cloacae* complex isolates. Thirteen DNA fragments were identified that could potentially serve as selective markers for the first clade, and 2 potential selective markers were detected for the second clade (sensitivity and specificity of 100%) (Table 4). Four DNA fragments were identified for cluster 1 (sensitivity of 100% and specificity of ≥96%) and four specific DNA fragments were identified for outbreak I isolates (sensitivity of ≥95% and specificity of ≥95%) (Table 4).

**Table 4:** Comparative results from the specific DNA fragments.

Identification no. DNA-fragment	Specificity (%)	DNA sequence comparative result^a^	Species	BLAST type	Identity (%)	E-value
first clade specific						
EnCl.031G9	100	partial operon for PQQ pyrroloquinoline quinone biosythesis	*Klebsiella pneumoniae* MGH 78578	nb	81	0
EnCl.012G4	100	partial pyrroloquinoline quinone	*Pseudomonas putida* F1	nb	70	−93
EnCl.025A10	100	nitrate reductase alpha-subunit	*Enterobacter* sp. 638	nb	89	0
EnCl.018F3	100	partial lactaldehyde dehydrogenase	*Enterobacter* sp. 638	nb	85	0
EnCl.012B11	100	TonB-dependent siderophore receptor	*Enterobacter* sp. 638	nb	85	0
EnCl.006A12	100	major facilitator superfamily MFS (efflux pump)	*Enterobacter* sp. 638	nb	79	0
EnCl.001D12	100	partial 1-acyl-sn-glycerol-3-phosphate acyltransferase	*Enterobacter* sp. 638	nb	82	−169
EnCl.032B2	100	putative helix-turn-helix, AraC type	*Klebsiella pneumoniae* MGH 78578	bx	56	−47
EnCl.030E5	100	hypothetical protein KPN_03581	*Klebsiella pneumoniae* MGH 78578	bx	66	−69
EnCl.020F8	100	protein tyrosine kinase	*Escherichia coli* O157:H7 EDL933	bx	62	−120
EnCl.011G11	100	amino-terminal domain similar to transcription regulators	*Listeria innocua* Clip11262	bx	26	−19
EnCl.005B7	100	response regulator receiver protein	*Enterobacter* sp. 638	bx	32	−3
EnCl.001C12	100	putative sulfatase	*Aeromonas salmonicida* A449	bx	66	−125
second clade specific						
EnCl.021G7	100	partial putative glutathione S-transferase	*Klebsiella pneumoniae* MGH 78578	nb	73	−89
EnCl.010E8	100	partial oxidoreductase alpha- (molybdopterin) subunit	*Enterobacter* sp. 638	nb	75	−134
cluster 1 specific						
EnCl.015H7	100	putative outer membrane usher protein	*Klebsiella pneumoniae* MGH 78578	bx	45	−100
EnCl.005F6	100	partial hypothetical protein	*Klebsiella pneumoniae* MGH 78578	nb	77	−129
EnCl.005F4	99	partial hypothetical protein	*Klebsiella pneumoniae* MGH 78578	nb	77	−128
EnCl.018G3	96	trypsin-like serine proteases	*Yersinia bercovieri* ATCC 43970	bx	63	−65
outbreak I specific						
EnCl.001G3	99	putative pili assembly chaperone	*Serratia proteamaculans* 568	bx	74	−54
EnCl.001D8	99	putative GTP-binding factor	*Escherichia coli* 536	nb	90	0
EnCl.027C11	97	hypothetical protein BL0477	*Bifidobacterium longum* NCC2705	bx	83	−35
EnCl.014H7	95	KilA N-terminal domain, N1R/P28 DNA binding	*Acanthamoeba polyphaga* mimivirus	bx	46	2

(For sequences see, Supplement Table S2)

a best hit in GenBank; nb: nucleotide-nucleotide blast; bx: nucleotide-protein blast (no hit with nucleotide-nucleotide blast).

## Discussion

The results of this study show that the CGH approach with a random library of 7 different strains (8 isolates) is a powerful method for studying new or poorly characterized species such as those that belong to the *E. cloacae* complex. Combined with MLSA, this technique can help to accurately assign isolates to species or subspecies. In this study, the CGH array had a high resolution because it was based on the presence or absence of 1,150 different genetic elements derived from different isolates, which were all evaluated on an individual basis. Therefore, CGH was able to determine genetic differences between different isolates and allowed unsupervised comparison of large sets of isolates. However, CGH had some limitations. One potential problem was the large number of mobile elements represented on the MGA. These mobile elements may bias genetic relationships between isolates. If these limitations are taken into account, the CGH approach can be used to determine the genetic relationship between (sub)species and, importantly, to identify specific markers for these (sub)species. Although, the *E. cloacae* complex is genetically heterogeneous, this study clearly shows that clinical isolates are evolutionary divided into two genetically distinct clades, where clade 1 appears to be younger than clade 2. Clade 1 isolates were mostly obtained from clinical settings with hospitalized patients, which is considered to be an environment with high specific (antibiotic) pressure, whereas the second clade contains a relatively large number of fecal isolates from patients without gastro-intestinal complaints.

Retrospectively, seven of the eight isolates used to construct the MGA belonged to the first clade. Therefore, the CGH results for the second clade should be interpreted with caution. After removal of the pQC sequences, which represented the most important mobile element present in the isolates, a similar clustering of isolates, with the exception of cluster 3, was obtained using either CGH or MLSA (Figure 2 and 4). The diversity of the second clade was larger when using MLSA than, but as mentioned previously, this may have been due to the limited number of isolates of this clade on the MGA. With CGH, specific genetic markers were identified that enabled differentiation between the two clades. In addition, three different genetic markers were identified that enabled specific identification of isolates belonging to cluster 1. Four different genetic markers were identified that allowed discrimination of outbreak I isolates from all tested isolates. MLSA was only performed on one outbreak isolate; therefore it is unknown whether all outbreak isolates cluster together after MLSA analysis. However, it is likely that such clustering occurs because of the clonality of the isolates. No specific genetic markers were detected for clusters of the second clade; this may have been due to the low number of isolates of clade 2 represented on the MGA. In the future, a small array could be constructed that contains specific markers for different (sub)species in order to correctly speciate isolates belonging to the *E. cloacae* complex. This new method to define and identify new species may be extended to other micro-organisms.

When combined, the MLSA and CGH methods have a synergetic effect. Where the CGH-array offers the advantage of being able to detect specific discriminating markers, MLSA is a standardized method whose results can be used for additional comparative studies. MLSA analysis divided the isolates into seven clusters. *Hsp60* and *rpoB* genotyping both did not sufficiently discriminate between the subspecies of *E. hormaechei*. Because CGH and MLSA also did also not discriminate between *E. hormaechei* subspecies, it is concluded that the current nomenclature for subspecies of *E. hormaechei* is inadequate. Furthermore, identification based on sequencing of *hsp60* and *rpoB* fragments did not always give identical outcomes. However, this was mostly based on differences in nomenclature between genotyping methods (e.g. *E. cloacae* IV / *E. kobei* and *E. hormaechei* / *E. cloacae* III). If differences in nomenclature are discarded, only 1.9% (3/158) of the isolates were misidentified based on sequencing of *hsp60* or *rpoB* fragments. Discrepancies arose for *E. asburiae*, *E. cloacae*, and an *E. hormaechei* according to *rpoB* genotyping, as well as *E. cloacae* III, *E. cloacae dissolvens*, and an *E. cloacae* IX according to *hsp60* genotyping (Table S1). For comparison, the Phoenix Automated Microbiology System (Becton Dickinson Biosciences) identified all isolates as *E. cloacae*. The present study demonstrates the superiority of genotypic identification over typical phenotypic identification for members of the *E. cloacae* complex. CGH and MLSA are clearly better methods for identifying isolates of the *E. cloacae* complex, but these methods are more expensive and labor-intensive. For diagnostic purposes, sequencing the 501 bp fragment of *rpoB* remains an acceptable alternative.

Correct speciation of isolates is clinically relevant. The current study showed that even when outbreak isolates were not included, isolates of the first clade were isolated nearly two times more often than isolates of the second clade. Furthermore, it is notable that more than half of the 13 fecal *E. cloacae* isolates from patients without gastro-intestinal complaints were found in the smaller second clade. Two of the DNA fragments specific for the first clade contained a partial operon for pyrroloquinoline quinone (pqq) biosythesis. Pqq is a co-factor of several dehydrogenases and transfers redox equivalents to the respiratory chain [25]. It has also been postulated that pqq reduces oxidative stress [26]. In addition, pqq stimulates bacterial growth [27]. A tempting but speculative hypothesis is that the isolates in the first clade are more pathogenic and isolates in the second clade are more commensal.

In conclusion, MLSA combined with CGH is a powerful method for studying species assignment within the *E. cloacae* complex. The complex is divided into two genetically distinct clades and consists of several more related and heterogenic (sub)species that must be redefined because of disagreements with known or proposed nomenclature. The CGH approach is a novel tool that could potentially be used to rapidly identify *Enterobacteriaceae* or even detect specific strains that threaten hospital populations.

## Materials and Methods

### Bacterial strains

One hundred twenty of the 158 isolates included in this study originated from the UMCU including 13 fecal isolates that were obtained from cultures taken from patients without gastro-intestinal complaints during their admission (Table S1). These 13 fecal isolates were considered commensal or transitional flora. These isolates were a subset of a large collection of isolates typed with Pulsed Field Gel Electrophoresis (PFGE) that represented the *E. cloacae* complex population in our hospital [28]. Twenty seven of the isolates belonged to PFGE genotype 1 (outbreak I), which is of great interest to us because this strain caused a nationwide outbreak and is now endemic in the UMCU [28]–[30]. Most isolates of PFGE genotype 1 contain a conjugative plasmid, pQC that carries the *qnrA1*, *bla*_CTX-M-9_, and *aadB* genes, which encode resistance to quinolones, extended-spectrum β-lactamases, and aminoglycosides, respectively, and thus provide this outbreak strain with a multidrug-resistant phenotype [30]. In addition, 21 isolates (10 outbreak I isolates) from 11 other Dutch hospitals were included. Also included were 12 isolates from 12 different European hospitals and 3 isolates from an industrial site. Finally, *E. cloacae* ATCC13047 and an ATCC 13047 conjugant with the pQC plasmid were included.

All isolates were previously identified as *E. cloacae* using an automated system and software (Phoenix Automated Microbiology System, Becton Dickinson Biosciences, Sparks, MD, U.S.). The *Enterobacter* species form a heterogenic group and its nomenclature is still undergoing alterations. Isolates identified with the Phoenix apparatus as *E. cloacae* were used as a starting point. When *hsp60* sequences indicated that an isolate did not belong to the genus *Enterobacter* it was excluded from the study.

In addition, *E. cloacae* ATCC 1307 with and without pQC was used for determining the contribution of DNA fragments on the microarray that were derived from this plasmid. Conjugation was performed as previously described [30]. All isolates were cultured overnight at 37°C on tryptic soy agar plates with 5% sheep blood. DNA was extracted with a NucleoSpin Tissue kit (Macherey-Nagel Gmbh & Co. KG, Düren, Germany) according to the manufacturer's instructions. DNA quality and quantity were measured with an ND-1000 Spectrophotometer (Wilmington, DE, U.S.) according to the manufacturer's instructions. A shotgun library was created from eight isolates (seven different strains). These eight isolates contained five isolates from the UMCU, two of which represented the outbreak strain (PFGE genotype 1) harboring the R-plasmid pQC (+/−300 kb), three isolates with different genetic backgrounds based on PFGE (including one with pQC), and three isolates that originated from different European countries (Table 3) [28], [30]. No high copy plasmid DNA was present in the DNA of the eight isolates used for the shotgun library.

### Detection of specific genes and genotyping

Target DNA for PCR was extracted by heating bacterial suspensions for 10 min at 95°C. Amplification of DNA fragments from *bla*_CTX-M-9_, *qnrA1*, *irp1*, *hsp60*, *fusA*, *gyrB*, *leuS*, *pyrG*, *rplB* and *rpoB* genes was performed with a Hotstart Taq MasterMix kit (Qiagen, Westburg b.v., Leusden, The Netherlands). Primers used, the size of the obtained products, relative primer positions, and annealing temperatures are described in Table S5. PCR products were purified with a Qiaquick PCR purification kit (Qiagen). Purified DNA was bidirectionally sequenced by BaseClear B.V. (Leiden, The Netherlands) according to their quick shot protocol (www.baseclear.com).

### Analysis of sequencing and genotyping results

Editing and analysis of chromatogram traces was performed using BioEdit [31]. Sequences of seven gene fragments (*hsp60*, *fusA*, *gyrB*, *leuS*, *pyrG*, *rplB* and *rpoB)* were confirmed by at least two chromatograms (forward and reverse). The quality of the phylogenetic information was determined by plotting transition and transversion rates as a function of the genetic distance calculated with a K80 model using DAMBE software [32], [33]. Pairwise nucleotide diversity (π), the number of segregating sides (θ), and tests for selection (Tajima's D, and Fu's F test) were calculated using DNAsp, version 4.20.2 [21], [22], [34]. The results showed (see results) that *rplB* is not suitable for phylogenetic analysis; thus *rplB* results were discarded for MLSA. ClonalFrame was used to assess the clonal relationship between *E. cloacae* complex isolates because we noticed high sequence diversity and putative recombination events in the six remaining gene fragments of the tested population. (http://www2.warwick.ac.uk/fac/sci/statistics/staff/research/didelot/clonalframe/). ClonalFrame determines the genetic relationships of bacteria based on point mutations and homologous recombination [35]. The burn-in length was 50,000 iterations and Markov chain Monte Carlo iterations were set at 50,000. After every 100 iterations, a posterior sample was recorded. Finally, a majority-ruled consensus tree was generated from all posterior samples. Congruence of the obtained tree was tested by phylogenic analyses of the concatenated sequences of the six gene fragments. The tree was constructed using maximum likelihood statistics with the “dnaml” program in PHYLIP. Parameter settings were: transition/transversion ratio = 2 based on data presented in Figure 1 and the transversion and transition rates as a function of the genetic distances, empirical base frequency, one category of rates, constant rate variation, and unweighted sites because sequence fragments are not expected to have different mutation frequencies and no selective pressure is expected (see results section). Prior to tree construction, 50.000 bootstraps of the data were performed using seqboot from the PHYLIP software package. Subsequently, the output was used to construct trees with SplitsTree4 (http://www.splitstree.org) [36].

### Microarray development

Equal amounts (10 µg) of chromosomal DNA from eight *E. cloacae* complex isolates (7 strains) were mixed to create a shotgun library as described by Borucki et al. (Table 3) [16], [37], [38]. For each isolate, equal amounts of genomic DNA were mixed, 10 µg of the pooled DNA were sonicated (Branson 250/450 Sonifier, 6 mm microtip, output intensity 1, Geneva, Switzerland) and fragments of approximately 1.2 kb were extracted from agarose gels (Qiaquick columns, Qiagen) and end-repaired (DNA Terminator End Repair Kit, Lucigen Corporation, Middleton, WI, U.S.). End-repaired fragments were ligated into the pSMART-HC-Kan vector (Clone-SMART, Lucigen). Subsequently, the recombinant plasmids were transformed into *E. coli* (ElectroMAX DH10B Cells, Invitrogen, Breda, The Netherlands) and plated on tryptone yeast plates with 30 µg/ml kanamycin. A total of 3,072 recombinant clones were arrayed into 96-well plates. Cloned DNA fragments were amplified by PCR using SMART primers (Lucigen) with 5′-C6 aminolinkers to facilitate cross-linking to the aldehyde-coated glass slides. PCR products were ethanol-purified and resuspended in 3×SSC (1×SSC: 0.15 M NaCl, 0.015 M sodium citrate, pH 7). All PCR products were printed on CSS silylated slides (European Biotech, Network, Dolembreux, Belgium) by an ESI three-axis DB-3 robot (ChipWriter Pro, Biorad, Hercules, CA, U.S.) at a controlled humidity of 55%. Slides were printed in four batches, after which they were blocked and denatured (15 sec dip in boiling water) following the manufacturer's instructions.

### Labeling, hybridization and data acquisition

Labeling, hybridization and data acquisition were performed as previously described by Leavis et al. [16]. A Bioprime system (Invitrogen) was used to label 0.5 µg DNA. For normalization, equal amounts of the 8 library isolates (7 strains) were mixed as the reference pool and labeled with Cy3 dUTP. Tester isolates were labeled with Cy5 dUTP. To test reproducibility, fifteen isolates were tested in duplicate, two in triplicate and one quadruplicate. For all hybridizations, Cy5 and Cy3 probes were combined with 100 µg yeast tRNA, dried with a Speedvac (30 min, at high temperature), resuspended in 40 µl Easyhyb buffer (Roche, Almere, The Netherlands), and denatured for 2 min at 100°C. Printed slides were pre-hybridized in 0.45 µm-filtered pre-hybridization buffer [1% BSA, 5×SSC, and 0.1% sodium dodecyl sulfate (SDS)] at 42°C for 45 min with rotation, then washed twice with purified water (MilliQ, Millipore, Billerica, MA, U.S.), dried with N_2_, and pre-warmed at 42°C. The hybridization mix was then pipetted on the printed side of the slide, covered with a hybrislib, placed in hybridization chambers (Corning Life Sciences B.V. Schiphol-Rijk, The Netherlands), and incubated overnight at 42°C in a water bath. Slides were then thoroughly washed sequentially in (a) 1×SSC, 0.2%SDS for 10 sec at 37°C, (b) 0.5×SSC for 10 sec at 37°C, and (c) twice in 0.2×SSC for 10 min at room temperature. Slides were dried with N_2_ and scanned using a Scanarray Express 680013 Microarray Analysis System (Perkin Elmers Life Analytical Sciences Inc., Wellesley, MA, U.S.). Images were obtained and quantified with ImaGene 4.2 software (Biodiscovery, Marina del Rey, CA, U.S.).

### Processing and analysis of MGA data

Inferior spots (empty spots or those when the Cy3 signal was less than 2 times the background), were excluded from normalization and data analysis [16]. Each slide was independently normalized to correct for individual differences. Cy5 minus background/Cy3 minus background ratios were calculated. Ratios were normalized by correcting for the overall signal intensities in both channels. Spots were selected for further analyses when 95% of the slides had a hybridization result for that spot. Next, the data were log2 transformed. The estimated probability of presence (EPP) of each insert was determined using a GACK-transformation [39]. (http://falkow.stanford.edu/whatwedo/software/software.html). With GACK-transformation, it is possible to dynamically choose cut-offs for grouping into present or divergent/absence of DNA-fragments based on the shape of the distribution. For hierarchical clustering and principal component analysis a graded output was selected. In our case the graded output generated a range of values from −0.5 to 0.5 in increments of 0.05, with −0.5 corresponding to 5% or less EPP and 0.5 corresponding to 95% or more EPP. The *E. cloacae* complex core genome was defined as genomic DNA fragments present in all isolates (GACK data>−0.5)[16]. For statistical analysis, the data were transformed to binary output, where 0 corresponded to 5% or less EPP, 1 to 95% or more EPP, and values in the 5%–95% interval were discarded. For parsimony analyze, the data were transformed into binary output, where 0 corresponded to <50% EPP, 1 to >50% EPP, and blanks were represented with a question mark. http://evolution.genetics.washington.edu/phylip.html [16], [40].

Complete linkage hierarchical clustering with Pearson correlation and principal component analysis (PCA) were performed and visualized with TIGR MeV version 3.1 software (http://www.tm4.org/mev.html). Data were also analyzed using Pars from the data package PHYLIP [40]. (http://evolution.genetics.washington.edu/phylip.html) Pars is a general parsimony program that performs the Wagner parsimony method with multiple states. Isolate 76 was chosen as the outgroup because this isolate appeared to be the most diverse after hierarchical clustering. A consensus tree was constructed from 50,000 trees. All analyses used all DNA fragments minus core and minus pQC-related DNA fragments. The output from the Pars analysis was used to construct trees using SplitsTree4 (http://www.splitstree.org) [36].

### Validation of the array

For each strain included in the shotgun library, the number of positive hybridizations (EPP >95%) was determined and gene coverage of the CGH array was calculated using a formula previously described by Akopyants et al. [24]: C_G_ = 1−(1−(T+I−2(RO/G)))^N^

where C_G_ = gene coverage, T = gene size, I = insert size, RO = required overlap and G = genome size. The average gene size was estimated to be 922 bp, which is the average gene length in *Enterobacter* sp. 638. The average length of DNA fragments spotted on the array was 1202 bp, based on sequences of 425 DNA fragments (data not shown). In contrast to oligonucleotide-based hybridizations, only overlaps between spotted DNA and test DNA are necessary for a random shotgun library based on DNA-DNA hybridization. The assumption was made that 100 nucleotides would be necessary for a positive hybridization result [24]. Finally, the size of the genomes of the *Enterobacter* isolates used to construct the CGH-array were estimated to be 4.5 million bp based on the whole genome sequences of *Enterobacter* sp. 638 and *Enterobacter sakazakii* ATCC BAA-894 (4,518,712 and 4,368,373 bp, respectively). Next, the minimal percentage of coverage of the genome was defined as:
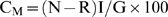
where C_M_ represents the minimal coverage of the core genome, N the number of DNA fragments on the MGA that passed quality control, R the redundancy (1/number of unique DNA fragments sequenced-1), I the DNA fragment size, and G the genome size [16]. To evaluate the robustness of hybridization, the presence or absence in the tested isolates of two DNA fragments located on two different bidirectionally sequenced DNA fragments was confirmed by PCR. The DNA fragments chosen were part of *irp1* encoding HMWP1 and part of *orf513* and *bla*_CTX-M-9_.

### Determination and exclusion of R-plasmid pQC-related DNA fragments

Since this study focused on studying the genetic relationships between species of the *E. cloacae* complex, interference by plasmid DNA should be excluded. The large size of pQC (+/−300 kb) prohibits its separation from chromosomal DNA. However, DNA fragments derived from pQC influence clustering analyses. Therefore, DNA fragments from pQC were removed for analyses. The paired student-T test (p<0.01) was used for three pairs of isolates to determine which DNA fragments were derived from pQC. Pairs of isolates were defined as isolates obtained from one patient with highly similar PFGE patterns (in our case all outbreak strains) where one isolate harbored pQC but the other did not. To verify pQC specificity, all isolates were hierarchically clustered using the 118 DNA fragments potentially derived from pQC. Twelve DNA fragments that were considered not to be specific for pQC were discarded. Isolates that tested positive on *qnrA1*-specific PCR were considered to carry pQC, as this gene is located on the plasmid [28], [30].

## Supporting Information

Table S1 Isolates used in this study
(0.05 MB XLS)

Table S2 Accession numbers of sequenced gene fragments
(0.04 MB XLS)

Table S3 Flag filtered, normalized 2log transferred data; * replicate slide
(6.85 MB XLS)

Table S4 Sequences of the sequenced DNA fragments used in the study
(0.06 MB XLS)

Table S5 Primers used for amplification and sequencing.
(0.02 MB XLS)
